# An exercise-based educational and motivational intervention after surgery can improve behaviors, physical fitness and quality of life in bariatric patients

**DOI:** 10.1371/journal.pone.0241336

**Published:** 2020-10-29

**Authors:** Francesca Gallé, Gianpaolo Marte, Assunta Cirella, Mirella Di Dio, Alessandra Miele, Roberta Ricchiuti, Fabrizio Liguori, Pietro Maida, Giorgio Liguori

**Affiliations:** 1 Department of Movement Sciences and Wellbeing, University of Naples “Parthenope”, Naples, Italy; 2 Evangelical Hospital “Villa Betania”, Naples, Italy; Weill Cornell Medical College in Qatar, QATAR

## Abstract

**Introduction:**

Unhealthy lifestyles may hinder bariatric surgery outcomes. This non-randomized controlled study aimed to evaluate the effects of an integrated post-operative exercise-based educational and motivational program in improving behaviors, quality of life, anthropometric features, cardiorespiratory and physical fitness in bariatric patients respect to the only surgical intervention.

**Methods:**

A group of adult sedentary bariatric patients chose to attend a 12-month exercise program integrated with diet education and motivational support, or to receive usual care. Dietary habits, binge eating disorder, physical activity, obesity-related quality of life, Body Mass Index, waist and hip circumference, VO_2_max, strength and flexibility were assessed at the start and at the end of the study in both groups.

**Results:**

On a total of 82 patients enrolled, follow-up measures were obtained from 28 (85.7% females, mean age 38.2±8.7) and 42 (71.4% females, mean age 40.2±9.5) patients included in the intervention and control group, respectively. All the behavioral and physical outcomes improved significantly in the participants to the intervention, while the control group showed lesser changes, especially regarding quality of life and physical fitness.

**Conclusions:**

Notwithstanding the self-selection, these results suggest that a timely postoperative behavioral multidisciplinary program for bariatric patients may be effective in establishing healthy behaviors which can lead to better surgery outcomes.

## Introduction

Currently, the epidemic of obesity represents a public health challenge and many efforts are required to control its determinants, mainly unhealthy diet and physical inactivity [1, 2]. Persons with obesity have an increased risk of developing chronic medical conditions, such as metabolic and cardiovascular diseases, and they are often affected by a lot of psychological and physical limitations [3]. In particular, the excess fat which is often associated with low levels of physical activity in these persons may result in reduced cardiorespiratory fitness, muscular strength and flexibility, with consequent low functional capacity to execute daily activities [4, 5]. Treatment of obesity includes pharmacologic and behavioral approaches, and surgery for morbid and severe cases [6]. Bariatric surgery is currently the most effective method to obtain sustained weight loss and remission or improvements of comorbid conditions, such as dyslipidemia, hypertension, type II diabetes, and obstructive sleep apnea [7–9]. However, the achievement and the durability of surgery outcomes may be hindered by psychosocial aspects or persisting unhealthy behaviors, leading to failure of primary interventions and following reoperations [10–12].

Several studies reported a high prevalence of psychological disorders and eating disorders in bariatric surgery candidates, which may be associated with greater difficulties in the adaptation to the new condition after surgery [13–15]. Literature shows that the identification and treatment of these disorders in the post-operative period can improve eating habits, surgery outcomes and patients’ quality of life [16–20].

In addition, the stability of weight loss after surgery may be influenced by behavioral factors such as compliance to the new nutritional regimen and to an active lifestyle, as recommended by guidelines [10, 11, 21, 22]. As for nutritional counselling, it was shown to be effective in attenuating weight regain after surgery [23].

Physical Activity (PA) is widely recognized as an effective instrument and recommended as an important component of multidisciplinary care programs aimed to improve psycho-physical conditions and surgery outcomes in bariatric patients [21–24]. In particular, exercise-based programs were shown to be successful in increasing weight loss, metabolic health, cardiorespiratory fitness and functional capacity after surgery [25–30]. However, exercise-based interventions are often focused exclusively on training to prevent weight regain and do not include motivational nor educational programs that may support patients in adopting new healthy behaviors, in order to guarantee the long-lasting maintenance of weight loss. Furthermore, little is known about the possible effects of these interventions in improving muscular strength and joint flexibility of bariatric patients beyond their weight condition [24].

Literature shows that, when implemented, exercise-based educational interventions are effective in improving weight loss maintenance in the post-operative period: therefore, structured multidisciplinary interventions including the contribute of various figures (surgeons, dieticians, psychologists, and movement experts) may provide to the patients the needed support and education to maximize the success of surgery [10, 29, 31–33]. However, these interventions are few, often performed several months after surgery, and they involve mainly patients who reported weight regain or a reduced weight loss [34–36]. Furthermore, similar multidisciplinary interventions are not routinely adopted in the clinical practice, as in Italy as in other countries.

The aim of the present study was to evaluate the effects of an integrated post-operative exercise-based educational and motivational program implemented immediately after surgery on lifestyles, quality of life, anthropometry, cardiorespiratory fitness, muscular strength and flexibility respect to the only surgical intervention in a sample of Italian sedentary bariatric patients.

## Methods

### Study design

This was a prospective, controlled and non-randomized study performed over the years 2016–2019 at the Evangelical Hospital Villa Betania in Naples. Participants were recruited progressively since November 2015 to March 2017; follow-up took place since March 2016 to January 2019.

The institutional review board of the Hospital Villa Betania approved the study protocol. The investigation was carried out in compliance with the ethical principles of the Helsinki Declaration for medical research involving human subjects. A written informed consent was obtained by all the participants and the anonymity of personal data was guaranteed. The study was registered to the ISRCTN registry with the identifier ISRCTN95430070.

### Setting and participants

Adult sedentary subjects who had undergone a first bariatric intervention no more than 6 months before the start of the intervention were considered. Individuals reporting any physical limitation due to underlying conditions such as cardiovascular or musculoskeletal (including arthritis) disorders, substance dependence, pregnancy, cognitive or psychiatric disorders were excluded. The eligible patients were invited to take part to the study in the hospital during the routine post-operative examination with the bariatric physician by a researcher who presented the aims and the design of the study. Those who accepted to participate in the study were not randomly allocated in the intervention or in the control group: patients who wanted to and could participate in the scheduled activities underwent the multidisciplinary intervention; those who did not want or were unable to take part in the proposed meetings because of other commitments, but were available to provide their clinical and physical information, underwent treatment as usual (TAU) and constituted the control group. TAU protocol consisted of a meeting with bariatric surgeon at 1 month and at 12 months after surgery. During these meetings patients received advice regarding diet and PA. They could see a dietitian upon request.

TAU patients were progressively matched to intervention patients in order to obtain at least a 2:1 ratio. Considering an expected difference in weight loss between groups of at least 14 kg [31], the recruitment of at least 6 patients was needed to obtain a power of 90% with a 95% CI.

### Intervention

The intervention included an exercise program, a dietary educational program, and motivational support. All these activities were free of charge and were provided for twelve months by the same staff in a hospital gym facility.

The exercise program consisted of 60-minute training sessions carried out two times per week and supervised by exercise specialists with expertise in adapted physical activity. Training protocols were developed on the basis of the American College of Sports Medicine guidelines for persons with obesity and tailored to participants’ conditions; the intensity of exercise was periodically increased according to the advances of the subjects [37]. Each session consisted of five phases: warm-up (10 min) including continuous walking or marching and exercises for joint activation; aerobic training (25 min) consisting of moderate and high-intensity brisk walking targeted at the level 4 (“somewhat strong”) of the 10-grade Borg’s Resting Perceived Exertion (RPE) scale or at 50–70% of maximum heart rate; exercises (15 min) to enhance strength of lower and upper limbs at 70–85% of repetition maximum (2 exercises, 3 sets of 12 repetitions); cool-down phase with agility/balance exercises (5 min) and flexibility static and dynamic exercises (5 min) [38].

The motivational program was carried out through periodical series of bi-weekly group meetings lasting 90 minutes and guided by a psychologist with expertise on motivational interviewing for behavior change. The first sessions were focused on the reciprocal introduction of participants and on their barriers, problems and readiness for behavior change; subsequently their thoughts, attitudes and beliefs regarding PA and diet were explored. In the course of the program, according to the social cognitive theory, patients were asked to set personally-meaningful goals, providing feedback, and exploring current and imagined futures regarding lifestyles in order to increase their psychological skills and enhance their self-efficacy [39]. The nutritional program was conducted through monthly group meetings lasting 90 minutes with a trained nutritionist. It was structured in a first phase aimed to investigate the nutritional habits of participants and in a subsequent intervention including the discussion of the effects of diet on weight management and the suggestion of healthy food choices and solutions to manage nutrition through an adequate daily distribution of meals and nutrients. In particular, the Mediterranean diet pattern was recommended as healthy eating model. Patients were encouraged to eat breakfast, to reduce intake of high energy density foods, and to identify individualized, short-term goals following the principles of the SMART (specific, measurable, attainable, realistic, and timely) goal setting. The importance of weight loss maintenance in order to improve both health and body image was also emphasized. Patient-centered behavior modification techniques such as self-monitoring, self-evaluation, goal setting, reinforcement, stimulus control, and relapse prevention were used in both programs [31, 33, 35].

TAU protocol for control patients consisted of periodical routine medical examinations by the surgeon. TAU patients were contacted by phone to participate to follow-up after 12 months since their recruitment.

### Outcomes

Sociodemographic features were collected for all the subjects who adhered to the study.

All the following outcomes were measured at the start (T_0_) and at the end (T_1_) of the 12-month intervention in participant and control patients in the same facility where the activities took place. All the measurements were performed by investigators who were blinded to the participants.

#### Behavioral outcomes and quality of life

Dietary patterns of participants were estimated by asking them to record the type and amount of food and beverage consumed for 7 days before T_0_ and T_1_. The frequency of food groups consumption was quantified in terms of number of servings per day (fruits, vegetables, cereals, and sweets) or week (meat, fish, dairy products, and eggs); the number of the days in a week patients consumed breakfast was also evaluated.

In order to explore eating behaviors and feeling/cognitions regarding binge eating episodes, we administered to both groups the 16-item Binge Eating Scale (BES), an instrument usually employed to assess the severity of binge eating among obese persons [40]. Three-four possible answers were proposed for each question, and a numerical value (0–3) was attributed to each of them. The presence of binge eating disorder (BED) was indicated by a BES cut-score of >17 (i.e., moderate/severe binge eating) or ≥27 (i.e., severe binge eating) [41].

The levels of habitual PA were assessed through the short format of the International Physical Activity Questionnaire (IPAQ) [42], which assesses the total energy expenditure per week by considering minutes spent on vigorous/moderate-intensity activities and walking. The IPAQ total score was expressed in MET-minutes/week.

The Obesity-Related WELL-being questionnaire (ORWELL-97) was administered to all the participants in order to detect possible variations in obesity-related quality of life and particularly to analyze their attitude towards PA [43]. The questionnaire included 18 items and is aimed to measure the importance attributed by subjects to a series of determinants related to quality of life (“relevance”) and the difficulties regarding these aspects that they actually perceived (occurrence) in relation to the overweight condition. The possible answers were “not at all”, “just a little”, “not so much” and “much”. A progressive number from 0 to 3 was attributed to each of these answers; the total score gave a measure of the quality of life in both participants and controls. A score in the ORWELL-97 questionnaire ≥ 70 was considered indicative of a clinically significant burden of obesity on quality of life [44].

#### Physical outcomes

Variations in BMI were investigated by measuring height and weight using a medical-certified scale and a stadiometer. Waist and hip circumference (WC, HC) were assessed using a non-stretchable tape and expressed in centimeters to the nearest 0.1 cm. WC was measured at the end of a normal expiration between the lowest border of rib cage and the upper border of iliac crest; HC was measured at the widest part of the hip, at the level of the greater trochanter.

Cardiorespiratory fitness was evaluated through the estimation of VO_2_max, which represents the highest amount of oxygen an individual can take in and utilize to produce ATP aerobically during exercise. The Rockport 1-mile walking test (RWT), which is the most commonly used field test to assess cardiorespiratory fitness and to predict aerobic fitness, was used to this aim [45]. Briefly, participants were asked to walk 1.6 km (one mile) as quickly as possible and their heart rate was collected via palpation immediately upon the completion of the path; the VO_2_max value was estimated through a formula that includes participants’ body weight, age, gender, time to complete one mile, and post-exercise heart rate [46]. VO_2_max was expressed as mL/kg^/^min.

The strength of lower limbs was evaluated through the chair squat test. Participants were asked to squat down from the standing position until they lightly touch a chair with their back. The number of repetitions performed by participants was considered as outcome [47]. A hydraulic hand dynamometer (Saehan-SH5005, Glanford Electronics Ltd, Scunthorpe, UK) was employed to assess muscle strength of upper limbs (grip strength). Three measurements were performed for each participant’s arm at each collection time; the mean obtained was expressed in Kg [37].

In order to detect possible changes in flexibility, participants’ passive Range of Motion (ROM) of shoulder, elbow, ankle and knee joints was assessed through a standard goniometer and expressed as degrees [48]. All joint motions were moved to their full extent and measured bilaterally; the joint RoM was measured to the nearest one degree.

### Statistical analyses

A descriptive analysis was carried out to evaluate the sociodemographic characteristics of the two groups at the start of the study. Age was expressed as mean value ± SD, while gender, education level and type of surgical intervention were expressed as number and percentage. Continuous variables were compared between groups through the Student’s *t* test for independent samples, while categorical variables were compared using the chi-square test.

The mean number of servings consumed per day or week ± SD for each food type and the mean number of breakfasts consumed per week ± SD were calculated for each group.

The mean BES values ± SD reached by intervention and TAU groups at the two times were calculated.

The mean IPAQ total score ± SD was calculated to express the medium level of habitual PA at the beginning and at the end of the study in both groups.

As for the quality of life, the mean total scores of the ORWELL-97 ± SD obtained from the two groups at the two times were calculated.

Physical outcomes measured at T_0_ and T_1_ for each group were reported as mean values ± SD.

All the variables were tested for normality through the Shapiro-Wilk test. Within-group changes from baseline to follow-up were analyzed using the Student’s *t* test for paired samples or the Wilcoxon Signed-Ranks test depending on the variables distribution. The comparisons between groups were carried out through the ANCOVA, using age, gender and baseline values of the dependent variable as covariates and including all their interaction terms in the models.

Statistical significance was declared at the 0.05 level. Statistical analyses were performed with the software IBM SPSS version 26 for Windows (Armonk, NY; IBM Corp., USA).

## Results

**Fig 1:**
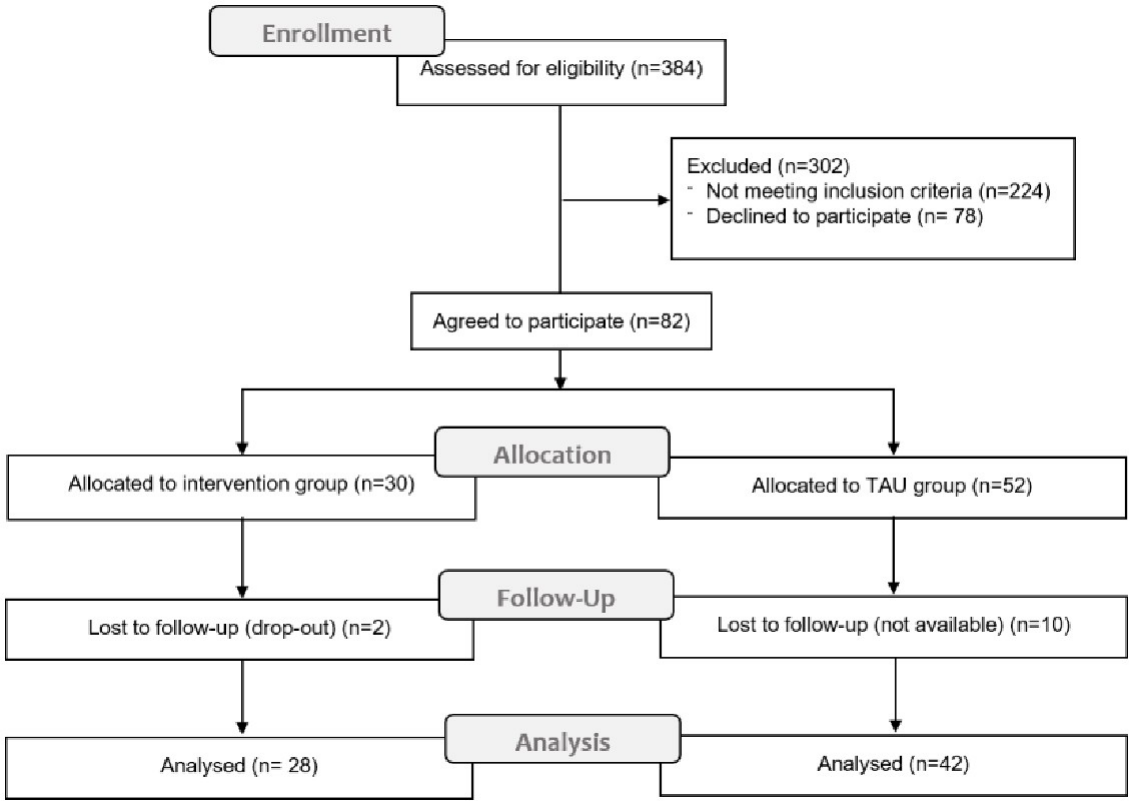
CONSORT flow-chart for enrollment, allocation, follow-up and analysis of participants to the study.

Fig 1 shows the flow-chart for the enrollment of participants to the investigation. On a total of 384 subjects who underwent a bariatric intervention in the considered period, 160 eligible patients were progressively invited to take part to the study. Of the 82 patients who accepted to participate, 30 constituted the intervention group and 52 composed the TAU group. The measurements of all the outcomes were obtained at the start of the study and at follow-up for 28 (85.7% females, mean age 38.2±8.7) and 42 (71.4% females, mean age 40.2±9.5) participants from the two groups respectively. The advanced reasons for dropping out at follow-up measurement were mainly logistic and organizational for individuals from both groups. No adverse events nor unintended effects were registered.

Age, sociodemographic characteristics and type of surgical intervention were not significantly different between the two final groups (Table 1).

**Table 1:** Baseline characteristics of intervention and control groups.

	Intervention (n = 28)	TAU (n = 42)	*p*
**Gender *n (%)***	24 F (85.7)	30 F (71.4)	0.27^a^
	4 M (14.3)	12 M (28.6)	
**Age *mean value ± SD***	38.2±8.7	40.2±9.5	0.37^b^
**Educational level *n (%)***			
• **Middle school**	8 (28.6)	10 (23.8)	0.34^a^
• **High school**	20 (71.4)	29 (60)	
• **University degree**	0 (0)	3 (7.1)	
**Type of surgery *n (%)***			
• **LSG**	18 (64.3)	23 (54.8)	0.83^a^
• **LAGB**	10 (35.7)	19 (45.2)	

TAU: Treatment As Usual; LSG: Laparoscopic Sleeve Gastrectomy; LAGB: Laparoscopic Adjustable Gastric Banding.

^a^ *χ*^*2*^ test.

^b^Student’s *t* test.

**Fig 2:**
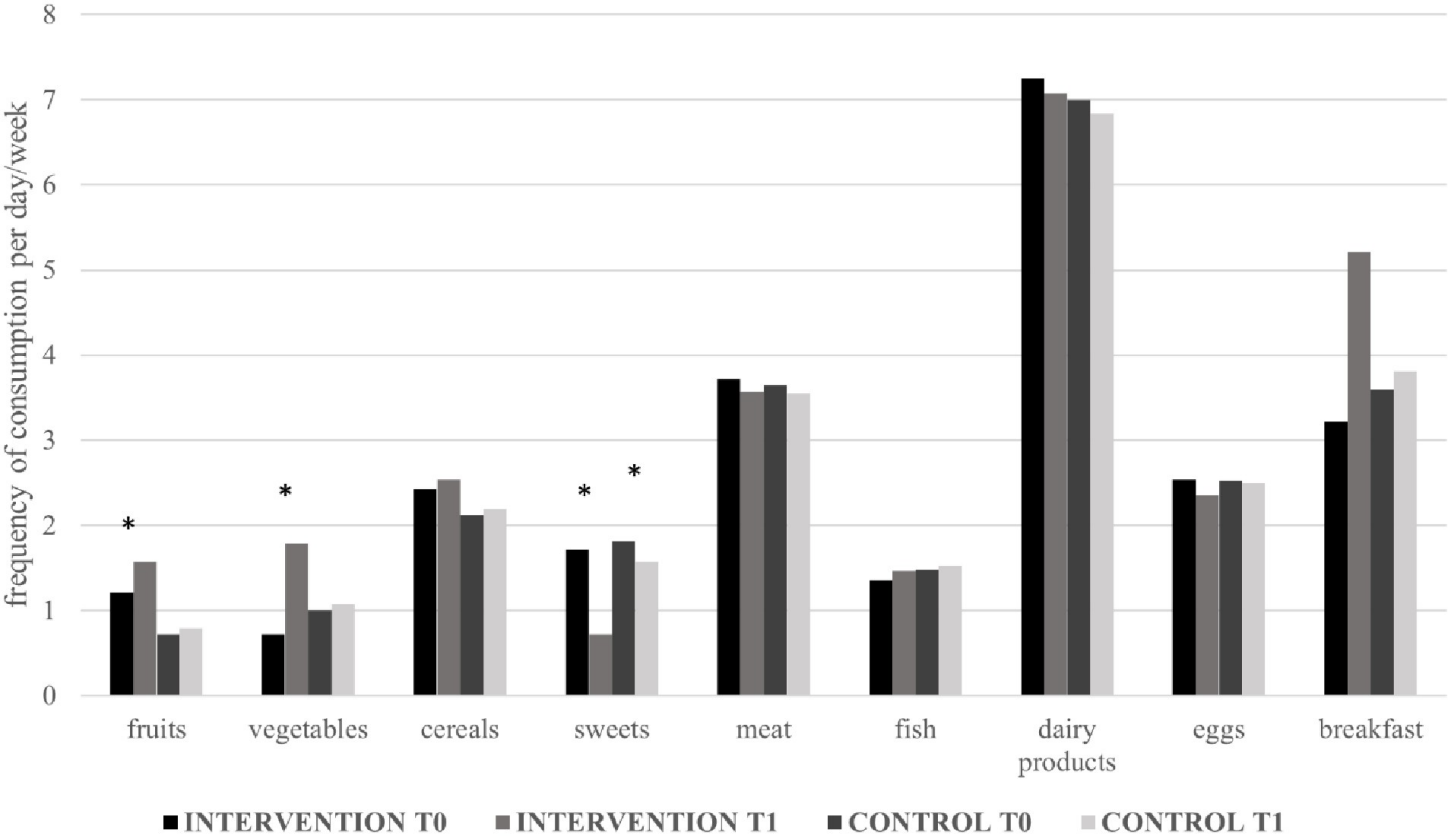
Dietary habits of participants to the intervention and controls at the start (T_0_) and at the end (T_1_) of the study. *Statistically significant difference between times from Student’s *t* test.

As for the diet, at the end of the intervention significant changes regarding fruits, vegetables, and sweets consumption were observed in the intervention group; TAU patients showed only a significant decrease in sweets consumption (Fig 2).

The presence of BED was diagnosed at the start of the study in the majority of the patients who accepted to participate to the intervention (4 with moderate BED, 20 with severe BED) and in all but two of the control patients (10 moderate, 30 severe). At the end of the study, only one among treated patients showed moderate BED, while 25 moderate and 10 severe BEDs were found among TAU patients (*p*<0.01). Table 2 shows the differences in mean BES values between the two groups at the two times.

**Table 2:** BES, IPAQ and ORWELL-97 mean values at T_0_ and T_1_ in both groups with related *p* and R squared values from ANCOVA.

	Intervention (n = 28)		TAU (n = 42)		*R*^*2*^	*p* (ANOVA)
Item mean ± SD	T_0_	T_1_	T_0_	T_1_		
**BES**	30.8±7.5	13.2±3.4*	29.8±4.8	20.4±7.5*	0.74	<0.01
**IPAQ** *(MET-min/week)*	439±86	652±53*	482±93	507±101*	0.85	<0.01
**ORWELL-97**	79±5.3	62.5±5.1*	80.1±5.7	79.8±5.9	0.89	<0.01

BES: Binge Eating Scale; IPAQ: International Physical Activity Questionnaire; ORWELL-97: Obesity-Related WELL-being questionnaire.

*Statistically significant intra-group difference between times

As for the IPAQ score, the increase of habitual PA levels reported by participants to the intervention at the end of the study was significantly higher than that of controls (Table 2).

Regarding the quality of life, at the start of the study quite all the patients enrolled perceived a high burden related to their weight status, showed by an ORWELL-97 score >70; at T_1_ only 3 patients from TAU group reported a score lower than 70, while all the patients but one who took part to the intervention showed a score under this threshold value. Table 2 shows the changes in the total mean scores obtained from ORWELL-97 questionnaire administered to participants to the intervention and TAU patients at the two collection times.

Table 3 shows the differences of the outcomes related to anthropometry, cardiorespiratory fitness, strength and flexibility measured at the start and at the end of the intervention in both groups with R^2^ and *p* values related to the comparisons between the two groups.

**Table 3:** Mean values ± SD of physical outcomes before (T_0_) and at the end (T_1_) of the intervention in participants and control patients with corresponding R^2^ and *p* values.

Outcome		T_0_ (mean ± SD)	T_1_ (mean ± SD)	Δ	*R*^*2*^	*p*
**BMI** (kg/m^2^)	**I**	33.8 ± 5.1	30.1 ± 3.8	-3.7*	0.69	<0.01
	**TAU**	33.3 ± 2.8	31.9 ± 5.3	-1.4*		
**WC** (cm)	**I**	105 ± 8.7	98.2 ± 7.5	-6.8*	0.85	<0.01
	**TAU**	104.8 ± 8.1	101.8 ± 8.2	-5*		
**HC** (cm)	**I**	103.5 ± 8	94.8 ± 8.1	-8.7*	0.81	<0.01
	**TAU**	103.1 ± 7.1	102 ± 7.7	-4.1		
**Estimated VO**_**2**_**max** (ml/kg^/^min)	**I**	20.2 ± 4.8	38.2 ± 5.4	18*	0.81	<0.01
	**TAU**	18.9 ± 10	23.3 ± 11.1	4.4*		
**Sit-and-stand** (n. of repetitions)	**I**	51.8 ± 21.2	98.8 ± 26.5	47*	0.96	<0.01
	**TAU**	49.2 ± 19.1	47.9 ± 13.6	-1.3		
**Grip strength** (kg)						
**right hand**	**I**	32.9 ± 10.5	49.2 ± 15.1	16.3*	0.98	<0.01
	**TAU**	32.8 ± 11.9	34 ±11.6	1.2*		
**left hand**	**I**	31.9 ±9.7	45.6 ± 13.4	13.7*	0.98	<0.01
	**TAU**	31.7 ± 11.3	31.9 ± 11.5	0.2*		
**Shoulder RoM** (degree)						
**extension**						
**right**	**I**	31.8 ± 4.8	41.9 ± 4.3	10.1*	0.99	<0.01
	**TAU**	32.5 ± 7.6	33.5 ± 7.6	1*		
**left**	**I**	31.5 ± 6.5	44.3 ± 4.8	12.8*	0.95	<0.01
	**TAU**	32.8 ± 5.1	33 ± 7.9	0.2		
**Elbow RoM** (degree)						
**extension**						
**right**	**I**	121.7 ± 22.4	153.8 ± 10.8	32.1*	0.88	<0.01
	**TAU**	122.3 ± 21.8	129.6 ± 21.5	7.3*		
**left**	**I**	125.6 ± 24.9	154.8 ± 16.5	29.2*	0.85	<0.01
	**TAU**	125.7 ± 25.9	127.9 ± 25.8	2.2		
**Ankle RoM** (degree)						
**flexion**						
**right**	**I**	71.8 ± 16.3	97.2 ± 14.8	25.4*	0.74	<0.01
	**TAU**	79.8 ± 10.8	83.4 ±10.8	3.6*		
**left**	**I**	71.4 ± 10.3	96.1 ± 12.6	24.7*	0.78	<0.01
	**TAU**	72.6 ± 11.2	76.6 ± 12.6	4*		
**extension**						
**right**	**I**	15.1 ± 3.9	19.9 ± 1.5	4.8*	0.94	<0.01
	**TAU**	13.7 ± 2.7	13.8 ± 2.9	0.1		
**left**	**I**	14.9 ± 4.1	19.9 ± 1.9	5*	0.68	<0.01
	**TAU**	14.4 ± 2.2	15.6 ± 3.9	1.2*		
**Knee RoM** (degree)						
**right**	**I**	187.2 ± 4.7	173.9 ± 3.9	-13.3*	0.69	<0.01
	**TAU**	186.9 ± 2.5	185.6 ± 4.8	-1.3		
**left**	**I**	186.8 ± 2.8	180.4 ± 3.7	-6.4*	0.48	<0.01
	**TAU**	186.7 ± 2.9	185.4± 3.3	-1.3		

I, Intervention group, n = 28; TAU, Treatment As Usual group, n = 42; Δ, differences between times; BMI: Body Mass Index; WC: Waist Circumference; HC: Hip Circumference; RoM: Range of Motion.

*Statistically significant intra-group difference between times

The considered variables showed a general improvement at the end of the year respect to baseline values in both groups, with the only exception of lower limbs strength in controls; however, the changes were more consistent and significant in the intervention group, especially for hip circumference and flexibility outcomes. The differences registered between groups were all significant.

## Discussion

This study was aimed to demonstrate the feasibility and the efficacy of an exercise-based program integrated with dietary education and motivational support in improving lifestyles, quality of life, anthropometric features and physical fitness of bariatric patients immediately after surgery.

Patients who chose and then participated to the nutritional intervention reported improved dietary patterns, consisting of an increased daily consumption of fruits and vegetables and a reduced use of sweets; the habit of having breakfast every day was also increased respect to the start of the intervention. Conversely, only a consistent reduction in sweets consumption was observed in control patients. This is in line with other studies which testify the need of structured educational interventions regarding diet rather than recommendations only to facilitate weight loss in bariatric patients [49, 50].

As for binging attitude, participants to the intervention showed a consistent improvement in BES and a remission of the disorder was observed in all but one of them, while controls did not reach these levels. This testifies that TAU patients did not increase their control and awareness about the amount of food, while patients who sought the educational/motivational intervention increased those psychological skills that are needed to change eating behaviors over the long term [35]. The diagnosis and treatment of eating disorders are fundamental to enhance and maintain the surgery effects: the best strategy is to educate patients regarding the amount, consistency and variety of their food in order to improve their nutrition, and to support them with cognitive behavioral strategies to reinforce healthy eating habits and encourage mindful eating. This can be done only through the integrated work of a health care team, where nutritionists and psychologists collaborate to support patients in their perioperative and postoperative path [51].

Quality of life improved significantly among the subjects who participated to the intervention, while no similar improvements were registered in the control group, suggesting that bariatric surgery alone may not be sufficient to this aim. These findings are in line with a systematic review which reported a strong evidence for the effects of psychosocial interventions on eating behaviors (e.g. binge eating) and psychological outcomes (e.g. quality of life) of bariatric patients [52].

Participants to the intervention increased also their levels of habitual PA. This confirms the effectiveness of an exercise-based motivational program in supporting sedentary individuals to adopt an active lifestyle, which is fundamental to maintain weight loss over time after surgery [24–27].

As for the physical outcomes, weight loss between the start and the end of the study was greater among participants to the intervention than among TAU patients. This allows to hypothesize that the behavioral changes induced by the lifestyle intervention were effective in preventing long-term weight regain. In fact, the most common causes of weight regain after bariatric surgery are the return to pre-operative eating habits, lack of exercise, or psychological issues [11]. Therefore, it is opportune that in the post-operative period bariatric patients receive personalized training programs integrated with health education on diet and exercise and a psychological support which can motivate them to change behavior and help them to address possible disorders [53, 54]. In this perspective, the motivational interviewing approach, by enhancing the autonomy of the patient in make behavior choices on the basis of his own motives with the collaboration of the therapist, may be effective in determining the durability of these programs [55]. With regards to this, it should be noted that the intervention described in this study was structured in group meetings and did not include personalized counselling. The use of individual sessions might have avoided possible patients’ inhibition to express their feelings to the group, leading to better results.

The intervention group showed also better improvements than controls in the other physical outcomes considered. These differences were registered both in anthropometric measures, which are directly related to weight loss, and in cardiorespiratory fitness, strength and flexibility, testifying the role of exercise in enhancing physical function of bariatric patients. The improvements registered among TAU patients testify the role of surgical intervention in improving the physical conditions of patients; however, the greater and significant changes shown by participants to the educational intervention suggest that an integrated post-operative support may further improve these aspects, as reported by other studies [24–29].

This study has some important limitations. First of all, the lack of randomization could have generated a selection bias in the constitution of the two patients’ groups, favoring the participation to the intervention by individuals more inclined for physical activity or better disposed to share their personal information. This consideration is also supported by the lower dropout registered among the participants to the intervention. The self-selection bias might have allowed to obtain a higher follow up rate and better outcomes in the intervention group than in the controls. However, it should be noted that many compliant subjects had to decline the invitation to participate to the proposed activities, or did not take part to the follow up measurements, due to their work or family duties. Moreover, since the exercise program was performed through group sessions and the proposed activities were not adapted to the characteristics of each participant, a consistent number of patients with certain cardiovascular conditions or musculoskeletal disorders requiring an individualized approach were excluded from the study. Unfortunately, these categories represented about the 70% and the 25% of the non-eligible patients respectively, and this would affect the external validity of the study. Consequently, the sample size was small and did not allow to analyze the outcomes with respect to the type of surgery. Considering that the magnitude and durability of the effects of Sleeve Gastrectomy and Laparoscopic Gastric Banding can be considerably different, this variable could have influenced the results [2]. Finally, the fitness tests used in this study were practical and likely to be used in clinical and fitness settings, but they were not highly objective and can be affected by pacing and failure to provide maximal effort, both of which would likely be improved in participants to the behavioral intervention. Therefore, it cannot be excluded that the improvements registered in the post- versus pre-intervention might not have been as large if more precise testing tools had been used.

Due to these limitations, the present investigation cannot allow to express definitive conclusions. In particular, the non-randomized design of the study limits the representativeness of our sample and the generalizability of our results. Further studies involving wider, randomized samples of patients are needed to confirm our findings.

However, the obtained results testify a general improvement in several aspects related to physical and psychological conditions of subjects who participated to the intervention. This indicates that the timely implementation of a structured program which integrates educational and motivational paths with exercise paths, providing patients with correct information regarding diet and physical activity, and supporting them in facing the physical and psychological changes following surgery, may be effective in establishing healthy behaviors which can lead to better surgery outcomes.

The early identification of wrong beliefs, lack of knowledge and possible psychological disorders which determine unhealthy lifestyles in obese patients seeking for bariatric surgery may be fundamental to address their post-operative behaviors through a comprehensive approach targeting diet, physical activity and psychological disorders. The review by Kalarchian and Marcus [56] and the systematic review by David et al. [52] indicated that the optimal time to initiate these interventions is soon after surgery, before significant weight regain has occurred [56]. In our study, patients were invited and directed to the activities at 1 month after surgery. This timing was probably fundamental in determining the benefits of the lifestyle intervention and should be considered in programs aimed to the prevention of weight regain after surgery. A follow-up study will be useful to evaluate the long-term stability of these effects.

## Conclusions

This study suggests that a timely post-operative lifestyle intervention may be useful for bariatric patients in order to help them in enhancing and maintaining surgery outcomes. As the recourse to bariatric surgery is becoming very common, it is fundamental that it could be considered a starting point for the establishment of a healthy lifestyle rather than an ending goal in obesity treatment. In this perspective, a multidisciplinary team composed by different professionals who sustain patients in adopting new behaviors may be fundamental in order to guarantee the long-term effectiveness of the surgical intervention. Adapting the post-operative support to the individual health and organizational needs of the patients may enhance their compliance. Due to the limitations related to the sample self-selection adopted in our investigation, further controlled randomized studies in this direction are needed.

## Supporting information

S1 Table Data from the intervention group at T_0_. (PDF)

S2 Table Data from the intervention group at T_1_. (PDF)

S3 Table Data from the control group at T_0_. (PDF)

S4 Table Data from the control group at T_1_. (PDF)

S1 Checklist TREND statement checklist. (PDF)

S1 File (PDF)

## References

[1] World Health Organization. Global strategy on diet, physical activity and health. WHO press, Geneva, 2004.

[2] Afshin A, Forouzanfar MH, Reitsma M, Sur P, Estep K, Murray CJ. Obesity and overweight and their health impact 1990–2015 in 195 countries. N Engl J Med. 2017; 377: 13–27. 28604169 10.1056/NEJMoa1614362 PMC5477817

[3] Hruby A, Hu FB. The epidemiology of obesity: a big picture. Pharmacoeconomics. 2015; 33(7): 673–689. 10.1007/s40273-014-0243-x 25471927 PMC4859313

[4] Park W, Ramachandran J, Weisman P, Jung ES. Obesity effect on male active joint range of motion. Ergonomics. 2010;53(1):102–8. 10.1080/00140130903311617 20069486

[5] Jeong Y, Heo S, Lee G, Park W. Pre-obesity and obesity impacts on passive joint range of motion. Ergonomics. 2018;61:9,1223–1231. 10.1080/00140139.2018.1478455 29775425

[6] Colquitt JL, Pickett K, Loveman E, Frampton GK. Surgery for weight loss in adults. Cochrane Database Syst Rev. 2014;8 10.1002/14651858.CD003641.pub4 25105982 PMC9028049

[7] Buchwald H, Avidor Y, Braunwald E, Jensen MD, Pories W, Fahrbach K, et al Bariatric surgery: a systematic review and meta-analysis. JAMA. 2004;292:1724–37. 10.1001/jama.292.14.1724 15479938

[8] Christou NV, Sampalis JS, Liberman M, Look D, Auger S, McLean AP, et al Surgery decreases long-term mortality, morbidity, and health care use in morbidly obese patients. Ann Surg. 2004;240:416–23. 10.1097/01.sla.0000137343.63376.19 15319713 PMC1356432

[9] Crémieux PY, Ledoux S, Clerici C, Cremieux F, Buessing M. The impact of bariatric surgery on comorbidities and medication use among obese patients. Obes Surg. 2010;20(7):861–70. 10.1007/s11695-010-0163-6 20440579

[10] Westerveld D, Yang D. Through thick and thin: identifying barriers to bariatric surgery, weight loss maintenance, and tailoring obesity treatment for the future. Surg Res Pract. 2016;2016:8616581 10.1155/2016/8616581 27314062 PMC4893581

[11] Mechanick JI, Youdim A, Jones DB, Garvey WT, Hurley DL, McMahon MM, et al Clinical practice guidelines for the perioperative nutritional, metabolic, and nonsurgical support of the bariatric surgery patient–2013 update: cosponsored by American Association of Clinical Endocrinologists, the Obesity Society, and American Society for Metabolic & Bariatric Surgery. Surg Obes Relat Dis. 2013;9(2):159–91. 10.1016/j.soard.2012.12.010 23537696

[12] Jensen MD, Ryan DH, Apovian CM, Ard JD, Comuzzie AG, Donato KA, et al 2013 AHA/ACC/TOS guideline for the management of overweight and obesity in adults: a report of the American College of Cardiology/American Heart Association Task Force on Practice Guidelines and The Obesity Society. J Am Coll Cardiol. 2014;63:2985–3023. 10.1016/j.jacc.2013.11.004 24239920

[13] Sarwer DB, Wadden TA, Fabricatore AN. Psychosocial and behavioral aspects of bariatric surgery. Obes Res 2005;13:639–48. 10.1038/oby.2005.71 15897471

[14] Pinto-Bastos A, Conceição EM, Machado PPP. Reoperative bariatric surgery: a systematic review of the reasons for surgery, medical and weight loss outcomes, relevant behavioral factors. Obes Surg 2017;27:2707–2715. 10.1007/s11695-017-2855-7 28791623

[15] Peterhänsel C, Wagner B, Dietrich A, Kersting A. Obesity and co-morbid psychiatric disorders as contraindications for bariatric surgery? A case study. Int J Surg Case Rep 2014;5:1268–70. 10.1016/j.ijscr.2014.11.023 25460490 PMC4275787

[16] Livhits M, Mercado C, Yermilov I, Parikh JA, Dutson E, Mehran A, et al Preoperative predictors of weight loss following bariatric surgery: systematic review. Obes Surg 2012;22:70–89. 10.1007/s11695-011-0472-4 21833817

[17] Kinzl JF. Morbid obesity: Significance of psychological treatment after bariatric surgery. Eat Weight Disord. 2010;15:275–80. 10.3275/7080 20513999

[18] Beck NN, Johannsen M, Støving RK, Mehlsen M, Zachariae R. Do postoperative psychotherapeutic interventions and support groups influence weight loss following bariatric surgery? A systematic review and meta-analysis of randomized and nonrandomized trials. Obes Surg 2012;22:1790–7. 10.1007/s11695-012-0739-4 22930073

[19] Gallé F, Cirella A, Salzano AM, Di Onofrio V, Belfiore P, Liguori G. Analyzing the effects of psychotherapy on weight loss after laparoscopic gastric bypass or laparoscopic adjustable gastric banding in patients with borderline personality disorder: a prospective study. Scand J Surg 2017;106:299–304. 10.1177/1457496917701670 28376683

[20] Gallé F, Maida P, Cirella A, Giuliano E, Belfiore P, Liguori G. Does post-operative psychotherapy contribute to improved comorbidities in bariatric patients with borderline personality disorder traits and bulimia tendencies? A prospective study. Obes Surg 2017;27:1872–1878. 10.1007/s11695-017-2581-1 28181141

[21] Garvey WT, Mechanick JI, Brett EM, Garber AJ, Hurley DL, Jastreboff AM, et al American Association of Clinical Endocrinologists and American College of Endocrinology comprehensive clinical practice guidelines for medical care of patients with obesity. Endocr Pract. 2016;22:1–203. 10.4158/EP161365.GL 27219496

[22] Società Italiana di Chirurgia dell’Obesità e delle malattie metaboliche (SICOB). Linee guida di chirurgia dell’obesità. SICOB 2016.

[23] Faria S.L., de Oliveira Kelly E., Lins R.D. Faria OP. Nutritional Management of Weight Regain After Bariatric Surgery. OBES SURG 20, 135–139 (2010). 10.1007/s11695-008-9610-z 18575942

[24] Bellicha A, Ciangura C, Poitou C, Portero P, Oppert JM. Effectiveness of exercise training after bariatric surgery-a systematic literature review and meta-analysis. Obes Rev. 2018;19(11):1544–1556. 10.1111/obr.12740 30156007

[25] Woodlief TL, Carnero EA, Standley RA, Distefano G, Anthony SJ, Dubis GS, et al Dose response of exercise training following Roux-en-Y gastric bypass surgery: a randomized trial. Obesity (Silver Spring). 2015;23(12):2454–61. 10.1002/oby.21332 26537198 PMC5480215

[26] Herring LY, Stevinson C, Carter P, Biddle SJH, Bowrey D, Sutton C, et al The effects of supervised exercise training 12–24 months after bariatric surgery on physical function and body composition: a randomised controlled trial. Int J Obes. 2017;41(6):909–16. 10.1038/ijo.2017.60 28262676

[27] Mundbjerg LH, Stolberg CR, Cecere S, Bladbjerg EM, Funch-Jensen P, Gram B, et al Supervised physical training improves weight loss after Roux-en-Y gastric bypass surgery: a randomized controlled trial. Obesity. 2018;26(5):828–837. 10.1002/oby.22143 29566463

[28] Castello V, Simões RP, Bassi D, Catai AM, Arena R, Borghi-Silva A. Impact of Aerobic Exercise Training on Heart Rate Variability and Functional Capacity in Obese Women After Gastric Bypass Surgery. Obes Surg 2011;21:1739–1749. 10.1007/s11695-010-0319-4 21104041

[29] Coen PM, Goodpaster BH. A role for exercise after bariatric surgery? Diabetes Obes Metab 2016;18(1):16–23. 10.1111/dom.12545 26228356 PMC5642115

[30] Tettero OM, Aronson T, Wolf RJ, Nuijten M, Hopman M, Janssen I. Increase in Physical Activity After Bariatric Surgery Demonstrates Improvement in Weight Loss and Cardiorespiratory Fitness. Obes Surg 2018;28:3950–3957. 10.1007/s11695-018-3439-x 30105664 PMC6223746

[31] Papalazarou A, Yannakoulia M, Kavouras SA, Komesidou V, Dimitriadis G, Papakonstantinou A, et al Lifestyle intervention favorably affects weight loss and maintenance following obesity surgery. Obesity 2010;18:1348–53. 10.1038/oby.2009.346 19834466

[32] Marshall S, Mackay H, Rich G, Isenring E. Do intensive preoperative and postoperative multidisciplinary interventions impact health-related bariatric surgery outcomes? A systematic review. Obes Surg 2019;29(S5), 231.

[33] Van Zyl N, Andrews L, Williamson H, Meyrick J. The effectiveness of psychosocial interventions to support psy-chological well-being in post-operative bariatric patients: A systematic review of evidence. Obes Res Clin Pract 2020, in press. 10.1016/j.orcp.2020.05.005 32631804

[34] Himes SM, Grothe KB, Clark MM, Swain JM, Collazo-Clavell ML, Sarr MG.: Stop regain: a pilot psychological intervention for bariatric patients experiencing weight regain. Obes Surg 2015;25:922–7. 10.1007/s11695-015-1611-0 25750006

[35] Bradley LE, Forman EM, Kerrigan SG, Swain JM, Collazo-Clavell ML, Sarr MG. A pilot study of an acceptance-based behavioral intervention for weight regain after bariatric surgery. Obes Surg 2016;26:2433–41. 10.1007/s11695-016-2125-0 26964997

[36] Bradley LE, Forman EM, Kerrigan SG, Goldstein SP, Butryn ML, Thomas JG, et al Project HELP: a remotely delivered behavioral intervention for weight regain after bariatric surgery. Obes Surg 2017;27:586–598. 10.1007/s11695-016-2337-3 27586525

[37] American College of Sports Medicine. ACSM’s Guidelines for Exercise Testing and Prescription 10^th^ ed Philadelphia, PA: Wolters Kluwer; 2017.

[38] Borg GA. Psychophysical bases of perceived exertion. Med Sci Sports Exerc. 1982; 14: 377–81. 7154893

[39] David LA, Sockalingam S, Wnuk S, Cassin SE. A pilot randomized controlled trial examining the feasibility, acceptability, and efficacy of Adapted Motivational Interviewing for post-operative bariatric surgery patients. Eat Behav. 2016;22:87–92. 10.1016/j.eatbeh.2016.03.030 27112113

[40] Gormally J, Black S, Daston S, Rardin D. The assessment of binge eating severity among obese persons. Addict Behav 1982;7:47–55. 10.1016/0306-4603(82)90024-7 7080884

[41] Grupski AE, Hood MM, Hall BJ, Azarbad L, Fitzpatrick SL, Corsica JA. Examining the Binge Eating Scale in screening for binge eating disorder in bariatric surgery candidates. Obes Surg 2013;23:1–6. 10.1007/s11695-011-0537-4 23104387 PMC4874644

[42] Craig CL, Marshall AL, Sjöström M, Bauman AE, Booth ML, Ainsworth BE, et al International physical activity questionnaire: 12-country reliability and validity. Med. Sci. Sports Exerc. 2003;35:1381–1395. 10.1249/01.MSS.0000078924.61453.FB 12900694

[43] Mannucci E, Ricca V, Barciulli E, Di Bernardo M, Travaglini R, Cabras PL, et al Quality of life and overweight: the obesity related well-being (Orwell 97) questionnaire. Addict Behav 1999;24:345–57. 10.1016/s0306-4603(98)00055-0 10400274

[44] Mannucci E, Petroni ML, Villanova N, Rotella CM, Apolone G, Marchesini G, et al Clinical and psychological correlates of health-related quality of life in obese patients. Health Qual Life Outcomes. 2010;8:90 10.1186/1477-7525-8-90 20731871 PMC2939642

[45] Kim K, Lee HY, Lee DY, Nam CW. Changes in cardiopulmonary function in normal adults after the Rockport 1 mile walking test: a preliminary study. J Phys Ther Sci. 2015;27(8):2559–61. 10.1589/jpts.27.2559 26356048 PMC4563314

[46] Kline GM, Porcari JP, Hintermeister R, Freedson PS, Ward A, McCarron RF, et al Estimation of VO_2_max from a one-mile track walk, gender, age, and body weight. Med Sci Sports Exerc. 1987, 19: 253–259. 3600239

[47] Government of Canada, Fitness and Amateur Sport. Canadian Standardized Test of Fitness, 1986 Operation Manual.

[48] Centers for Disease Control and Prevention. 2010. “Public Use Dataset for Normal Joint Range of Motion.” October 27. Accessed 5 January 2020. http://www.cdc.gov/ncbddd/jointrom/

[49] Nijamkin MP, Campa A, Sosa J, Baum M, Himburg S, Johnson P. Comprehensive nutrition and lifestyle education improves weight loss and physical activity in Hispanic Americans following gastric bypass surgery: a randomized controlled trial. J Acad Nutr Diet 2012; 112:382–90. 10.1016/j.jada.2011.10.023 22717198

[50] Kalarchian MA, Marcus MD, Courcoulas AP, Lutz C, Cheng Y, Sweeny G. Structured dietary intervention to facilitate weight loss after bariatric surgery: A randomized, controlled pilot study. Obesity 2016;24:1906–12. 10.1002/oby.21591 27466039

[51] McGrice M, Don Paul K. Interventions to improve long-term weight loss in patients following bariatric surgery: challenges and solutions. Diabetes Metab Syndr Obes Target Ther 2015;8:263–274. 10.2147/DMSO.S57054 26150731 PMC4485844

[52] David LA, Sijercic I, Cassin SE. Preoperative and post-operative psychosocial interventions for bariatric surgery patients: A systematic review. Obes Rev. 2020 10.1111/obr.12926 31970925

[53] Richardson WS, Plaisance AM, Periou L, Buquoi J, Tillery D. Long-term Management of Patients After Weight Loss Surgery. Ochsner J. 2009;9(3):154–9. 21603433 PMC3096273

[54] Jassil FC, Manning S, Lewis N, Steinmo S, Kingett H, Lough F, et al Feasibility and Impact of a Combined Supervised Exercise and Nutritional-Behavioral Intervention following Bariatric Surgery: A Pilot Study. J Obes. 2015;2015:693829 10.1155/2015/693829 26199740 PMC4493296

[55] Hardcastle SJ, Taylor AH, Bailey MP, Harley RA, Hagger MS. Effectiveness of a motivational interviewing intervention on weight loss, physical activity and cardiovascular disease risk factors: a randomised controlled trial with a 12-month post-intervention follow-up. Int J Behav Nutr Phys Act. 2013;10:40 10.1186/1479-5868-10-40 23537492 PMC3639183

[56] Kalarchian MA, Marcus MD. Psychosocial interventions pre and post bariatric surgery. Eur Eat Disord Rev 2015;23:457–62. 10.1002/erv.2392 26364715

