# Supplementary material for: An exercise-based educational and motivational intervention after surgery can improve behaviors, physical fitness and quality of life in bariatric patients
Source: PLoS One. 2020 Oct 29;15(10):e0241336. doi: 10.1371/journal.pone.0241336 (PMC7595397; doi:10.1371/journal.pone.0241336)
Supplement: S1 Table — (PDF) [file pone.0241336.s001.pdf]

ID	GENDER	AGE	WEIGHT	HEIGHT	BMI	WC	HC	VO2 MAX	SQUAT	UPPER LIMB STRENGHT RIGHT	UPPER LIMB STRENGHT LEFT	SHOULDER ROM EXTENSION RIGHT	SHOULDER ROM EXTENSION LEFT	ELBOW ROM EXTENSION RIGHT	ELBOW ROM EXTENSION LEFT	ANKLE ROM FLEXION RIGHT	ANKLE ROM FLEXION LEFT	ANKLE ROM EXTENSION RIGHT	ANKLE ROM EXTENSION LEFT	KNEE ROM RIGHT	KNEE ROM LEFT	BES	IPAQ	ORWELL	FRUITS/DAY	VEGETABLES/DAY	CEREALS/DAY	SWEETS/DAY	MEAT/WEEK	FISH/WEEK	MILK, YOGURT AND DAIRY PRODUCTS/WEEK	EGGS/WEEK	BREAKFAST/WEEK
1	F	28	139	1,63	52,3	122	120	19,8	21	15	15	29	27	100	100	63	65	14	10	180	185	32	288	79	2	0	2	1	5	1	7	0	0
2	M	37	141	1,68	50,0	120	118	28,3	90	49	46	38	41	156	177	74	68	15	14	187	187	31	312	69	2	2	2	2	5	0	2	1	0
3	F	42	90	1,57	36,5	119	115	22,7	34	19	19	25	23	100	100	88	84	18	18	189	189	32	356	76	1	0	3	1	3	2	5	2	2
4	F	35	78	1,6	30,5	92	85	20,2	40	35	34	34	37	127	127	75	70	20	20	194	191	39	537	82	1	0	3	2	4	1	5	4	5
5	M	29	97	1,72	32,8	104	103	26,8	99	49	46	38	39	150	150	70	72	11	12	185	185	31	476	75	3	1	2	2	1	1	0	4	0
6	M	33	95	1,76	30,7	97	95	30,6	93	45	44	37	38	123	120	79	75	12	16	180	184	37	253	81	2	2	3	2	6	1	13	3	7
7	F	34	78	1,58	31,2	110	103	25,6	47	34	32	35	35	143	143	75	70	15	15	188	188	40	367	69	0	0	2	2	3	0	2	2	0
8	F	35	82	1,54	34,6	112	110	19,8	55	35	35	34	36	120	120	45	55	9	10	180	185	30	513	80	1	0	3	1	4	2	3	3	0
9	F	32	78	1,61	30,1	90	89	17,9	64	46	45	39	39	143	156	55	62	7	10	184	184	38	487	80	0	1	2	2	4	3	10	5	5
10	F	28	84	1,6	32,8	110	103	20,6	78	36	35	34	36	100	100	98	91	14	15	186	186	32	512	76	1	1	1	2	4	1	5	2	0
11	F	53	78	1,58	31,2	95	98	24,7	62	32	31	34	37	100	100	60	70	19	22	195	190	39	523	82	1	0	3	1	2	2	15	0	7
12	M	33	125	1,85	36,5	115	108	24,4	37	47	45	37	38	177	170	95	90	20	20	194	186	38	458	91	2	1	2	2	3	3	14	1	7
13	F	39	80	1,56	32,9	105	105	18,3	95	38	37	40	41	154	150	87	84	19	19	188	188	34	378	84	1	0	3	2	5	1	8	0	5
14	F	37	88	1,69	30,8	96	95	19,7	44	36	35	32	29	127	127	80	69	8	8	182	182	38	567	81	1	1	2	2	3	2	5	6	0
15	F	41	84	1,62	32,0	105	105	21,3	35	34	34	32	32	140	150	63	60	13	13	195	187	41	497	78	1	0	3	1	4	1	10	2	7
16	F	48	80	1,59	31,6	102	103	16,8	54	38	36	33	31	100	110	97	86	18	19	191	191	31	506	75	2	2	3	2	5	1	5	3	5
17	F	53	80	1,58	32,0	105	104	21,5	47	37	35	34	29	106	105	70	70	16	15	188	188	26	479	69	1	0	2	1	2	3	5	2	0
18	F	45	87	1,65	32,0	104	104	23,6	38	36	35	35	37	115	120	55	66	19	17	190	190	26	342	81	0	1	3	2	3	1	6	2	3
19	F	43	87	1,6	34,0	110	105	19,8	41	35	34	34	34	135	159	74	70	18	18	186	186	25	317	77	2	0	3	2	5	1	15	2	7
20	F	24	78	1,57	31,6	97	103	17,9	47	16	16	30	32	149	155	90	80	22	19	190	187	26	425	79	0	2	2	2	4	2	5	5	0
21	F	36	83	1,58	33,2	108	103	20,6	32	20	20	25	25	110	110	60	65	12	8	179	179	33	446	84	3	1	3	2	4	1	14	3	7
22	F	43	90	1,62	34,3	111	115	21,4	37	19	19	27	25	102	100	50	60	12	12	189	183	34	483	80	0	1	2	2	3	2	5	4	4
23	F	34	79	1,57	32,0	94	103	11	50	16	16	27	23	100	100	65	72	16	16	186	186	33	534	75	1	0	3	2	3	2	18	1	7
24	F	40	82	1,65	30,1	90	90	20,5	61	37	35	30	32	124	147	86	80	18	18	190	190	32	521	92	1	1	2	1	2	1	5	3	5
25	F	52	87	1,63	32,7	105	103	12,7	44	15	15	25	23	100	100	45	50	10	8	180	188	17	448	82	3	1	1	2	5	0	6	2	0
26	F	57	85	1,6	33,2	110	105	13,7	37	35	34	24	21	100	110	55	66	15	11	188	188	16	340	77	1	1	2	1	4	1	3	2	0
27	F	24	90	1,65	33,1	108	104	10,5	38	35	34	27	23	102	100	98	85	18	19	190	190	15	487	81	0	0	3	2	3	1	2	4	0
28	F	35	91	1,66	33,0	104	103	15,8	30	37	35	25	22	105	110	58	65	16	16	187	187	16	439	79	1	1	3	2	5	1	10	3	7

S1 Table. Data from the Intervention Group at T_0.
