# Supplementary material for: An exercise-based educational and motivational intervention after surgery can improve behaviors, physical fitness and quality of life in bariatric patients
Source: PLoS One. 2020 Oct 29;15(10):e0241336. doi: 10.1371/journal.pone.0241336 (PMC7595397; doi:10.1371/journal.pone.0241336)
Supplement: S2 Table — (PDF) [file pone.0241336.s002.pdf]

ID	GENDER	AGE	WEIGHT	HEIGHT	BMI	WC	HC	VO2 MAX	SQUAT	UPPER LIMB STRENGHT RIGHT	UPPER LIMB STRENGHT LEFT	SHOULDER ROM EXTENSION RIGHT	SHOULDER ROM EXTENSION LEFT	ELBOW ROM EXTENSION RIGHT	ELBOW ROM EXTENSION LEFT	ANKLE ROM FLEXION RIGHT	ANKLE ROM FLEXION LEFT	ANKLE ROM EXTENSION RIGHT	ANKLE ROM EXTENSION LEFT	KNEE ROM RIGHT	KNEE ROM LEFT	BES	IPAQ	ORWELL	FRUITS/DAY	VEGETABLES/DAY	CEREALS/DAY	SWEETS/DAY	MEAT/WEEK	FISH/WEEK	MILK, YOGURT AND DAIRY PRODUCTS/WEEK	EGGS/WEEK	BREAKFAST/WEEK
1	F	28	117	1,63	44,0	112	115	25,6	25	25	20	39	42	145	145	115	115	18	19	176	178	15	654	54	2	2	3	1	4	1	7	0	7
2	M	37	119	1,68	42,2	111	108	32,3	157	73	59	48	50	167	177	110	110	19	19	175	179	13	635	49	2	2	3	0	4	1	5	1	7
3	F	42	75	1,57	30,4	108	107	35,7	76	29	28	37	38	146	130	102	110	18	18	174	180	14	619	59	1	2	3	1	3	2	5	2	7
4	M	35	75	1,6	29,3	90	85	44,3	81	50	48	44	48	160	160	85	85	20	20	171	179	13	726	67	1	2	3	0	4	1	7	4	7
5	M	29	91	1,72	30,8	100	94	45,0	146	73	66	48	49	162	167	115	100	18	18	174	180	14	626	63	3	2	2	2	1	1	0	4	0
6	F	33	88	1,76	28,4	90	84	44,7	128	74	63	47	50	157	157	110	110	19	20	176	180	14	615	65	2	2	3	0	6	1	13	3	7
7	F	34	69	1,58	27,6	98	94	44,5	88	51	46	44	50	143	145	83	89	18	18	171	178	26	624	59	1	2	2	2	3	1	2	2	3
8	F	35	69	1,54	29,1	106	101	40,2	95	50	49	45	48	154	164	75	75	19	19	172	181	13	712	60	1	1	3	1	4	2	3	3	7
9	F	32	76	1,61	29,3	86	81	44,7	104	67	60	49	50	158	174	78	85	19	20	171	181	16	612	63	1	2	2	0	3	3	10	3	5
10	F	28	78	1,6	30,5	102	100	35,6	132	49	49	43	49	145	130	104	102	20	20	181	192	12	758	58	1	1	3	0	4	1	5	2	7
11	F	53	69	1,58	27,6	90	85	45,3	102	42	41	44	49	141	140	78	80	22	22	170	180	12	713	63	1	2	3	0	2	2	15	0	7
12	M	33	109	1,85	31,8	112	104	38,7	98	75	66	46	49	178	185	105	100	23	23	172	182	13	608	65	2	2	2	0	3	3	14	1	7
13	F	39	70	1,56	28,8	98	95	38,5	153	60	54	50	51	175	175	111	102	21	21	175	185	8	602	71	1	2	3	0	5	1	8	0	5
14	F	37	80	1,69	28,0	90	90	39,1	95	52	49	42	43	145	145	100	100	20	20	182	183	9	743	68	2	2	2	2	3	2	5	4	0
15	F	41	78	1,62	29,7	100	97	32,1	93	44	44	42	45	174	174	89	90	20	20	169	180	11	610	63	1	1	3	0	4	1	10	2	7
16	F	48	73	1,59	28,9	97	96	26,3	94	58	54	43	41	143	146	116	112	22	22	174	179	10	756	65	2	2	3	0	5	1	5	3	7
17	F	53	73	1,58	29,2	100	97	39,4	88	57	55	44	42	143	145	86	85	20	20	172	180	14	611	59	1	2	2	1	2	3	5	2	0
18	F	45	78	1,65	28,7	96	96	39,4	98	46	45	42	47	156	156	105	106	22	23	181	190	13	628	67	2	2	3	0	3	1	6	2	7
19	F	43	76	1,6	29,7	100	97	32,4	84	45	46	44	45	158	170	98	101	20	18	176	179	12	612	67	2	1	3	0	4	2	10	2	7
20	F	24	70	1,57	28,4	91	88	41,3	89	36	30	40	45	160	160	110	110	23	21	179	178	11	645	69	1	2	2	2	4	2	5	4	0
21	F	36	71	1,58	28,4	98	94	41,8	87	39	34	35	40	142	142	115	100	22	22	174	179	10	638	55	2	2	3	0	4	1	14	3	7
22	F	43	76	1,62	29,0	101	95	37,1	85	33	30	37	40	159	159	80	80	19	17	168	174	16	633	61	2	2	2	1	3	2	5	4	7
23	F	34	70	1,57	28,4	87	83	40,2	90	23	20	36	39	157	157	77	77	18	16	176	180	17	684	59	1	2	3	0	3	2	9	1	7
24	F	40	80	1,65	29,4	88	84	40,3	123	58	56	39	42	159	179	112	100	20	23	180	184	9	748	67	1	1	2	1	3	1	5	3	7
25	F	52	78	1,63	29,4	100	98	30,7	97	23	20	38	37	140	120	79	80	20	20	170	179	12	598	69	3	2	1	2	4	0	6	2	0
26	F	57	75	1,6	29,3	105	99	35,7	87	48	47	36	38	145	140	89	90	19	21	169	178	13	613	63	2	1	2	2	4	1	7	2	7
27	F	24	78	1,65	28,7	97	95	40	89	50	49	37	41	148	145	116	115	18	17	171	179	15	621	65	2	2	2	1	3	1	2	4	0
28	F	35	80	1,66	29,0	96	91	38,4	83	53	50	38	39	147	147	80	83	20	20	170	175	16	625	58	1	2	3	1	5	1	10	3	7

S2 Table. Data from the Intervention Group at T_1.
