# Supplementary material for: An exercise-based educational and motivational intervention after surgery can improve behaviors, physical fitness and quality of life in bariatric patients
Source: PLoS One. 2020 Oct 29;15(10):e0241336. doi: 10.1371/journal.pone.0241336 (PMC7595397; doi:10.1371/journal.pone.0241336)
Supplement: S3 Table — (PDF) [file pone.0241336.s003.pdf]

ID	GENDER	AGE	WEIGHT	HEIGHT	BMI	WC	HC	VO2 MAX	SQUAT	UPPER LIMB STRENGHT RIGHT	UPPER LIMB STRENGHT LEFT	SHOULDER ROM EXTENSION RIGHT	SHOULDER ROM EXTENSION LEFT	ELBOW ROM EXTENSION RIGHT	ELBOW ROM EXTENSION LEFT	ANKLE ROM FLEXION RIGHT	ANKLE ROM FLEXION LEFT	ANKLE ROM EXTENSION RIGHT	ANKLE ROM EXTENSION LEFT	KNEE ROM RIGHT	KNEE ROM LEFT	BES	IPAQ	ORWELL	FRUITS/DAY	VEGETABLES/DAY	CEREALS/DAY	SWEETS/DAY	MEAT/WEEK	FISH/WEEK	MILK, YOGURT AND DAIRY PRODUCTS/WEEK	EGGS/WEEK	BREAKFAST/WEEK
1	F	60	97	1,60	37,9	121	121	9,4	43	15	15	22	23	121	121	73	73	12	13	192	190	35	545	78	0	1	1	2	4	0	2	2	2
2	F	39	79	1,50	35,1	110	111	41,2	76	19	18	24	23	93	93	68	57	10	10	191	190	30	490	81	2	1	2	2	3	1	2	2	0
3	F	49	109	1,65	40,0	118	116	41,0	30	19	18	26	27	111	111	82	71	13	14	185	185	34	544	76	1	1	2	2	3	1	4	2	2
4	M	45	118	1,70	40,8	123	120	10,4	38	49	47	42	36	161	165	91	90	20	16	186	186	33	511	88	0	2	2	2	5	2	7	2	4
5	F	27	125	1,76	40,4	117	115	12,7	31	29	28	26	31	129	134	93	80	14	14	187	184	29	528	87	3	1	1	2	2	1	1	3	1
6	M	44	106	1,76	34,2	100	95	22,4	39	48	46	41	37	155	164	90	85	16	13	180	182	28	540	75	0	0	2	1	3	2	15	3	6
7	M	46	102	1,77	32,6	95	91	11,5	47	50	47	46	41	133	137	73	60	11	13	187	185	25	489	67	0	2	3	2	5	3	3	2	3
8	F	45	105	1,62	40,0	122	110	12,5	32	35	35	38	36	152	160	91	91	16	15	186	180	32	548	79	0	2	3	2	6	0	7	3	4
9	F	47	78	1,55	32,5	104	100	22,0	52	15	15	25	30	147	147	87	85	10	13	184	183	33	557	70	1	1	3	1	3	2	14	2	7
10	F	37	82	1,54	34,6	112	103	14,6	34	22	22	20	21	127	132	95	88	13	13	192	190	31	551	76	0	1	1	1	2	1	3	4	2
11	M	49	115	1,81	35,1	107	98	24,7	44	46	44	41	40	92	92	65	61	10	12	184	184	24	558	83	0	2	3	1	2	1	10	2	5
12	M	24	78	1,61	30,1	95	98	40,7	91	51	47	43	39	171	184	78	65	12	20	181	183	30	540	75	0	1	1	3	3	3	7	2	5
13	M	43	102	1,77	32,6	98	100	21,6	42	49	46	40	37	134	134	96	80	17	16	187	187	26	579	81	1	0	1	2	4	1	10	3	7
14	F	56	92	1,63	34,6	112	100	11,0	36	25	25	28	38	142	145	83	69	16	17	184	183	32	535	69	1	1	3	2	3	3	12	5	7
15	M	35	97	1,78	30,6	98	100	39,0	98	48	48	41	37	119	119	90	83	13	13	185	186	23	558	85	0	1	2	2	5	1	10	2	7
16	F	38	87	1,59	34,4	109	110	9,7	39	26	26	27	28	95	95	64	60	14	15	190	190	35	212	81	0	1	3	2	2	1	8	3	5
17	F	45	81	1,60	31,6	100	101	10,0	38	28	28	32	34	127	137	67	65	15	16	184	187	34	388	76	1	0	3	1	3	1	0	2	0
18	F	30	90	1,60	35,2	116	111	10,2	43	30	30	28	29	98	98	65	60	13	13	187	187	25	312	82	0	1	4	1	3	1	15	3	7
19	F	39	70	1,50	31,1	102	103	38,7	66	20	20	26	29	106	110	80	75	15	14	188	188	36	356	91	1	1	3	1	5	1	8	2	4
20	F	39	85	1,65	31,2	101	100	10,3	34	27	27	30	32	111	114	73	70	12	15	187	187	26	546	84	1	2	3	2	5	1	3	2	3
21	F	48	87	1,60	34,0	103	102	11,6	37	33	32	35	34	146	150	70	65	15	17	185	186	33	356	81	1	2	2	3	4	1	10	2	5
22	F	27	93	1,66	33,7	113	115	11,4	51	31	30	27	29	94	94	82	70	13	14	187	188	25	459	84	0	1	2	2	3	1	5	3	3
23	F	32	84	1,60	32,8	98	99	23,7	39	34	32	33	37	140	165	85	63	12	12	186	191	25	367	87	0	0	2	1	3	2	9	4	4
24	F	26	90	1,63	33,9	108	107	10,3	36	34	32	39	40	123	126	75	63	11	11	187	190	31	514	85	3	1	3	2	3	2	7	2	5
25	M	45	93	1,70	32,2	98	98	10,1	55	39	39	37	37	121	121	90	76	16	16	191	193	35	487	81	2	2	2	1	5	0	3	2	0
26	F	37	85	1,55	35,4	108	105	23,3	52	29	28	30	32	153	156	85	84	17	15	188	188	38	512	79	1	1	1	1	4	1	3	2	3
27	M	37	92	1,68	32,6	98	101	21,6	78	49	48	40	36	125	130	91	91	18	18	186	189	34	523	80	0	1	1	3	3	2	2	1	0
28	F	49	93	1,61	35,9	115	110	11,5	44	16	15	25	28	112	115	88	80	15	15	188	188	33	458	82	1	1	2	2	5	4	13	2	5
29	M	23	97	1,81	29,6	98	97	25,6	92	55	52	46	39	145	169	87	90	16	20	189	188	35	378	90	0	0	2	3	4	3	12	3	5
30	F	43	92	1,65	33,8	102	103	10,3	42	19	19	25	29	97	95	63	60	10	11	186	187	31	567	86	0	0	1	2	3	2	6	2	3
31	F	52	92	1,63	34,6	108	105	11,3	36	16	16	25	31	115	120	65	65	13	15	185	180	33	497	78	1	2	1	3	5	1	2	3	0
32	F	35	81	1,65	29,8	98	100	22,6	37	35	35	34	37	119	122	91	91	20	16	187	185	30	506	75	1	1	2	2	3	1	9	1	3
33	F	38	87	1,59	34,4	103	104	10,8	61	15	15	21	23	96	94	64	61	13	15	186	187	35	479	69	1	0	2	2	5	0	11	2	7
34	F	48	85	1,60	33,2	98	100	9,0	38	35	34	41	39	127	132	93	85	17	17	190	191	29	342	81	0	2	1	2	4	2	3	2	1
35	F	59	89	1,63	33,5	105	102	18,9	34	35	32	32	32	121	120	83	75	15	15	189	186	25	237	78	0	1	2	1	3	2	15	3	7
36	F	39	81	1,58	32,4	96	97	24,0	44	21	21	24	27	91	89	64	58	14	14	187	187	26	438	79	1	0	3	2	5	2	5	3	5
37	M	34	92	1,75	30,0	94	91	22,4	87	53	50	42	37	148	155	78	70	15	15	188	187	30	484	84	1	1	3	2	3	1	7	4	3
38	F	46	90	1,68	31,9	97	98	26,7	42	26	25	28	30	134	137	80	65	10	14	186	183	29	534	80	1	1	2	1	4	0	10	2	5
39	M	27	100	1,75	32,7	98	96	26,3	90	50	48	44	33	123	127	90	78	14	15	188	188	16	567	75	2	1	3	2	5	2	3	3	0
40	F	47	90	1,64	33,5	105	103	9,2	35	34	33	26	33	92	90	93	75	11	11	189	185	32	634	89	1	1	2	2	3	1	3	3	1
41	F	35	92	1,68	32,6	97	95	27,5	47	38	35	39	35	95	90	65	57	10	13	187	187	30	524	83	0	1	1	2	2	2	3	3	0
42	F	23	86	1,61	33,2	100	101	10,9	38	37	34	31	33	97	93	67	58	9	11	189	189	17	482	75	1	0	3	1	3	3	12	3	5

S3 Table. Data from the Control Group at T_0.
