# Supplementary material for: An exercise-based educational and motivational intervention after surgery can improve behaviors, physical fitness and quality of life in bariatric patients
Source: PLoS One. 2020 Oct 29;15(10):e0241336. doi: 10.1371/journal.pone.0241336 (PMC7595397; doi:10.1371/journal.pone.0241336)
Supplement: S4 Table — (PDF) [file pone.0241336.s004.pdf]

| ID | GENDER | AGE | WEIGHT | HEIGHT | BMI | WC | HC | VO2 MAX | SQUAT | UPPER LIMB STRENGHT RIGHT | UPPER LIMB STRENGHT LEFT | SHOULDER ROM EXTENSION RIGHT | SHOULDER ROM EXTENSION LEFT | ELBOW ROM EXTENSION RIGHT | ELBOW ROM EXTENSION LEFT | ANKLE ROM FLEXION RIGHT | ANKLE ROM FLEXION LEFT | ANKLE ROM EXTENSION RIGHT | ANKLE ROM EXTENSION LEFT | KNEE ROM RIGHT | KNEE ROM LEFT | BES | IPAQ | ORWELL | FRUITS/DAY | VEGETABLES/DAY | CEREALS/DAY | SWEETS/DAY | MEAT/WEEK | FISH/WEEK | MILK, YOGURT AND DAIRY PRODUCTS/WEEK | EGGS/WEEK | BREAKFAST/WEEK |
|---|---|---|---|---|---|---|---|---|---|---|---|---|---|---|---|---|---|---|---|---|---|---|---|---|---|---|---|---|---|---|---|---|---|
| 1 | F | 60 | 98 | 1,60 | 38,3 | 117 | 120 | 14,5 | 44 | 18 | 15 | 24 | 23 | 121 | 121 | 73 | 73 | 12 | 13 | 190 | 190 | 30 | 545 | 79 | 0 | 1 | 1 | 2 | 4 | 0 | 2 | 2 | 2 |
| 2 | F | 39 | 75 | 1,50 | 33,3 | 107 | 107 | 40,5 | 71 | 20 | 19 | 25 | 23 | 109 | 105 | 68 | 57 | 10 | 10 | 179 | 190 | 21 | 490 | 80 | 2 | 2 | 2 | 2 | 3 | 1 | 2 | 2 | 0 |
| 3 | F | 49 | 110 | 1,65 | 40,4 | 118 | 116 | 41,5 | 42 | 20 | 18 | 27 | 26 | 111 | 111 | 82 | 71 | 13 | 14 | 192 | 185 | 31 | 544 | 76 | 1 | 1 | 2 | 2 | 3 | 1 | 4 | 2 | 2 |
| 4 | M | 45 | 115 | 1,70 | 39,8 | 110 | 118 | 13,7 | 39 | 49 | 48 | 42 | 42 | 154 | 165 | 91 | 90 | 20 | 22 | 186 | 186 | 33 | 511 | 75 | 0 | 2 | 2 | 2 | 5 | 2 | 7 | 2 | 4 |
| 5 | F | 27 | 135 | 1,76 | 43,6 | 117 | 118 | 15,8 | 35 | 30 | 28 | 27 | 25 | 130 | 134 | 93 | 80 | 14 | 14 | 191 | 184 | 18 | 528 | 87 | 3 | 1 | 2 | 2 | 2 | 1 | 1 | 3 | 1 |
| 6 | M | 44 | 103 | 1,76 | 33,3 | 98 | 96 | 23,0 | 40 | 49 | 46 | 42 | 42 | 155 | 164 | 100 | 100 | 20 | 22 | 180 | 182 | 19 | 540 | 76 | 0 | 0 | 2 | 1 | 3 | 2 | 12 | 3 | 6 |
| 7 | M | 46 | 96 | 1,77 | 30,6 | 95 | 93 | 18,9 | 48 | 51 | 48 | 47 | 46 | 133 | 137 | 73 | 60 | 11 | 13 | 190 | 185 | 18 | 489 | 67 | 0 | 2 | 3 | 2 | 5 | 3 | 3 | 2 | 3 |
| 8 | F | 45 | 115 | 1,62 | 43,8 | 121 | 114 | 11,9 | 33 | 37 | 35 | 39 | 38 | 152 | 160 | 96 | 91 | 18 | 20 | 179 | 180 | 21 | 548 | 80 | 0 | 2 | 3 | 2 | 5 | 0 | 7 | 3 | 4 |
| 9 | F | 47 | 80 | 1,55 | 33,3 | 107 | 103 | 23,4 | 53 | 17 | 15 | 26 | 26 | 147 | 147 | 87 | 85 | 10 | 13 | 184 | 183 | 18 | 557 | 70 | 1 | 2 | 3 | 1 | 3 | 2 | 14 | 2 | 7 |
| 10 | F | 37 | 80 | 1,54 | 33,7 | 110 | 100 | 15,2 | 35 | 23 | 22 | 21 | 21 | 127 | 132 | 105 | 100 | 13 | 15 | 185 | 190 | 19 | 551 | 75 | 0 | 1 | 2 | 1 | 2 | 1 | 3 | 4 | 2 |
| 11 | M | 49 | 88 | 1,81 | 26,9 | 100 | 96 | 38,4 | 45 | 47 | 44 | 42 | 41 | 165 | 140 | 88 | 61 | 10 | 12 | 181 | 184 | 9 | 635 | 85 | 0 | 2 | 3 | 1 | 2 | 1 | 10 | 2 | 5 |
| 12 | M | 24 | 70 | 1,61 | 27,0 | 94 | 98 | 40,1 | 80 | 52 | 48 | 45 | 45 | 187 | 184 | 78 | 65 | 12 | 15 | 179 | 183 | 18 | 540 | 70 | 0 | 1 | 1 | 0 | 3 | 3 | 7 | 2 | 5 |
| 13 | M | 43 | 100 | 1,77 | 31,9 | 98 | 99 | 22,7 | 43 | 49 | 46 | 41 | 41 | 134 | 134 | 96 | 80 | 17 | 18 | 181 | 187 | 20 | 764 | 81 | 1 | 0 | 1 | 2 | 4 | 1 | 10 | 3 | 7 |
| 14 | F | 56 | 87 | 1,63 | 32,7 | 103 | 99 | 12,7 | 37 | 27 | 26 | 30 | 30 | 142 | 140 | 83 | 69 | 16 | 17 | 184 | 183 | 22 | 535 | 69 | 1 | 1 | 3 | 2 | 3 | 3 | 10 | 5 | 7 |
| 15 | M | 35 | 65 | 1,78 | 20,5 | 94 | 89 | 43,8 | 80 | 49 | 48 | 42 | 42 | 129 | 125 | 90 | 83 | 13 | 13 | 179 | 186 | 6 | 754 | 85 | 0 | 1 | 2 | 2 | 5 | 1 | 10 | 2 | 7 |
| 16 | F | 38 | 86 | 1,59 | 34,0 | 102 | 110 | 13,2 | 40 | 27 | 26 | 28 | 27 | 95 | 95 | 64 | 60 | 14 | 15 | 187 | 190 | 30 | 356 | 81 | 0 | 1 | 3 | 2 | 2 | 1 | 8 | 3 | 5 |
| 17 | F | 45 | 87 | 1,60 | 34,0 | 98 | 104 | 13,8 | 39 | 29 | 28 | 33 | 33 | 127 | 127 | 67 | 65 | 15 | 16 | 184 | 185 | 33 | 388 | 83 | 1 | 0 | 3 | 1 | 3 | 1 | 0 | 2 | 0 |
| 18 | F | 30 | 95 | 1,60 | 37,1 | 115 | 113 | 11,8 | 44 | 31 | 30 | 28 | 27 | 98 | 98 | 70 | 60 | 13 | 13 | 187 | 187 | 20 | 409 | 86 | 0 | 1 | 3 | 1 | 3 | 1 | 15 | 2 | 7 |
| 19 | F | 39 | 70 | 1,50 | 31,1 | 100 | 104 | 35,6 | 67 | 21 | 19 | 27 | 27 | 129 | 129 | 99 | 88 | 15 | 16 | 191 | 188 | 31 | 356 | 90 | 1 | 1 | 3 | 1 | 4 | 1 | 8 | 2 | 4 |
| 20 | F | 39 | 60 | 1,65 | 22,0 | 93 | 96 | 18,9 | 42 | 28 | 27 | 33 | 31 | 134 | 140 | 73 | 65 | 12 | 15 | 188 | 187 | 21 | 546 | 84 | 1 | 2 | 3 | 2 | 3 | 1 | 3 | 2 | 3 |
| 21 | F | 48 | 86 | 1,60 | 33,6 | 101 | 101 | 13,4 | 38 | 34 | 33 | 36 | 35 | 146 | 159 | 76 | 63 | 15 | 17 | 191 | 186 | 19 | 356 | 81 | 1 | 2 | 2 | 1 | 4 | 1 | 10 | 2 | 5 |
| 22 | F | 27 | 91 | 1,66 | 33,0 | 99 | 106 | 15,4 | 52 | 33 | 30 | 28 | 28 | 100 | 90 | 90 | 84 | 13 | 14 | 183 | 181 | 9 | 459 | 85 | 1 | 1 | 2 | 2 | 3 | 1 | 5 | 3 | 3 |
| 23 | F | 32 | 85 | 1,60 | 33,2 | 100 | 102 | 25,9 | 40 | 35 | 32 | 35 | 37 | 140 | 140 | 85 | 80 | 12 | 12 | 180 | 180 | 18 | 367 | 85 | 1 | 1 | 2 | 1 | 3 | 2 | 9 | 4 | 4 |
| 24 | F | 26 | 60 | 1,63 | 22,6 | 97 | 98 | 32,8 | 35 | 36 | 33 | 41 | 41 | 149 | 160 | 75 | 78 | 11 | 11 | 193 | 190 | 20 | 514 | 81 | 3 | 1 | 3 | 2 | 3 | 2 | 7 | 2 | 5 |
| 25 | M | 45 | 92 | 1,70 | 31,8 | 95 | 98 | 15,4 | 38 | 40 | 39 | 38 | 37 | 121 | 121 | 96 | 93 | 16 | 20 | 185 | 180 | 32 | 487 | 81 | 2 | 2 | 2 | 1 | 5 | 0 | 3 | 2 | 0 |
| 26 | F | 37 | 70 | 1,55 | 29,1 | 100 | 99 | 30,2 | 50 | 30 | 29 | 31 | 32 | 158 | 163 | 90 | 84 | 17 | 21 | 188 | 188 | 30 | 512 | 68 | 1 | 1 | 1 | 1 | 4 | 1 | 3 | 2 | 3 |
| 27 | M | 37 | 90 | 1,68 | 31,9 | 97 | 99 | 13,4 | 69 | 49 | 48 | 42 | 41 | 145 | 147 | 96 | 91 | 18 | 18 | 192 | 189 | 18 | 523 | 80 | 0 | 1 | 1 | 0 | 3 | 2 | 2 | 1 | 0 |
| 28 | F | 49 | 91 | 1,61 | 35,1 | 115 | 107 | 12,2 | 45 | 20 | 16 | 26 | 24 | 126 | 126 | 90 | 92 | 15 | 17 | 181 | 188 | 19 | 458 | 82 | 1 | 1 | 2 | 2 | 5 | 4 | 13 | 2 | 5 |
| 29 | M | 23 | 90 | 1,81 | 27,5 | 95 | 98 | 33,7 | 81 | 54 | 52 | 46 | 45 | 156 | 156 | 87 | 84 | 16 | 20 | 190 | 188 | 19 | 405 | 90 | 0 | 0 | 2 | 1 | 4 | 3 | 12 | 3 | 5 |
| 30 | F | 43 | 93 | 1,65 | 34,2 | 108 | 105 | 15,7 | 40 | 20 | 19 | 26 | 27 | 102 | 90 | 77 | 73 | 10 | 10 | 183 | 187 | 20 | 567 | 86 | 0 | 0 | 2 | 2 | 3 | 2 | 6 | 2 | 3 |
| 31 | F | 52 | 89 | 1,63 | 33,5 | 107 | 104 | 19,8 | 45 | 19 | 16 | 25 | 24 | 120 | 120 | 75 | 70 | 13 | 15 | 192 | 180 | 33 | 497 | 78 | 1 | 2 | 2 | 3 | 5 | 1 | 2 | 3 | 0 |
| 32 | F | 35 | 90 | 1,65 | 33,1 | 103 | 104 | 23,0 | 38 | 36 | 35 | 35 | 35 | 121 | 120 | 95 | 91 | 20 | 23 | 193 | 185 | 20 | 506 | 79 | 1 | 1 | 2 | 2 | 3 | 1 | 9 | 2 | 3 |
| 33 | F | 38 | 86 | 1,59 | 34,0 | 103 | 103 | 15,1 | 62 | 16 | 15 | 22 | 21 | 108 | 98 | 66 | 61 | 13 | 15 | 180 | 180 | 30 | 479 | 76 | 1 | 0 | 2 | 2 | 5 | 0 | 11 | 2 | 7 |
| 34 | F | 48 | 82 | 1,60 | 32,0 | 95 | 101 | 11,5 | 39 | 36 | 34 | 41 | 41 | 127 | 132 | 93 | 84 | 17 | 17 | 180 | 180 | 19 | 342 | 81 | 0 | 2 | 1 | 2 | 4 | 2 | 3 | 2 | 1 |
| 35 | F | 59 | 88 | 1,63 | 33,1 | 105 | 102 | 19,4 | 35 | 36 | 32 | 33 | 32 | 121 | 120 | 83 | 78 | 15 | 23 | 191 | 186 | 9 | 367 | 78 | 0 | 1 | 2 | 1 | 3 | 2 | 13 | 3 | 7 |
| 36 | F | 39 | 75 | 1,58 | 30,0 | 95 | 97 | 27,1 | 45 | 21 | 21 | 25 | 24 | 95 | 81 | 72 | 62 | 14 | 14 | 182 | 182 | 11 | 438 | 79 | 1 | 0 | 3 | 2 | 5 | 3 | 5 | 3 | 5 |
| 37 | M | 34 | 71 | 1,75 | 23,2 | 88 | 87 | 41,2 | 70 | 52 | 51 | 43 | 42 | 157 | 163 | 78 | 70 | 15 | 21 | 181 | 187 | 19 | 578 | 84 | 1 | 1 | 3 | 2 | 3 | 2 | 7 | 3 | 3 |
| 38 | F | 46 | 62 | 1,68 | 22,0 | 88 | 86 | 43,7 | 43 | 27 | 24 | 29 | 26 | 135 | 120 | 80 | 80 | 10 | 14 | 186 | 183 | 21 | 534 | 80 | 1 | 1 | 2 | 1 | 4 | 0 | 10 | 2 | 5 |
| 39 | M | 27 | 85 | 1,75 | 27,8 | 94 | 98 | 36,7 | 65 | 49 | 48 | 44 | 43 | 123 | 100 | 90 | 88 | 14 | 19 | 188 | 188 | 6 | 567 | 70 | 2 | 1 | 3 | 2 | 5 | 2 | 3 | 3 | 3 |
| 40 | F | 47 | 82 | 1,64 | 30,5 | 100 | 101 | 11,7 | 40 | 35 | 33 | 26 | 26 | 98 | 90 | 93 | 90 | 11 | 10 | 193 | 190 | 18 | 768 | 89 | 1 | 1 | 2 | 2 | 3 | 1 | 3 | 3 | 5 |
| 41 | F | 35 | 75 | 1,68 | 26,6 | 95 | 95 | 38,4 | 48 | 37 | 35 | 40 | 40 | 115 | 95 | 70 | 60 | 10 | 9 | 187 | 187 | 19 | 524 | 83 | 1 | 1 | 1 | 2 | 2 | 2 | 3 | 3 | 2 |
| 42 | F | 23 | 85 | 1,61 | 32,8 | 98 | 100 | 12,1 | 39 | 37 | 35 | 32 | 32 | 100 | 90 | 71 | 60 | 9 | 9 | 180 | 189 | 9 | 482 | 75 | 1 | 0 | 3 | 1 | 3 | 3 | 12 | 3 | 5 |

# S4 Table. Data from the Control Group at $T_1$.
