# Supplementary material for: An exercise-based educational and motivational intervention after surgery can improve behaviors, physical fitness and quality of life in bariatric patients
Source: PLoS One. 2020 Oct 29;15(10):e0241336. doi: 10.1371/journal.pone.0241336 (PMC7595397; doi:10.1371/journal.pone.0241336)
Supplement: S1 File — (PDF) [file pone.0241336.s006.pdf]

Project: Multidisciplinary path of nutritional education, motivational support and adapted physical activity as a therapeutic tool in obese subjects undergoing bariatric surgery

Cattedra di Igiene ed Epidemiologia, Dipartimento di Scienze Motorie e del Benessere, Università di Napoli "Parthenope"

Background

Obesity is a multifactorial condition: genetic, environmental and individual factors contribute to determine an alteration of caloric intake and energy use, with consequent excessive fat accumulation. Worldwide, the prevalence of obesity has doubled since 1980. In 2014, more than 11% of adult men and 15% of women were obese. In 2013, more than 42 millions children aged ≤5 years were overweight.

In 2014, more than on third of the adult Italian population (36,1%) was overweight, and one person out of ten (10,3%) was obese (http://dati.istat.it/).

Obesity increases the risk of diabetes, hypertension, cardiovascular diseases and some type of cancer, and has important socio-economic costs. The European Chart on Fighting Obesity Actions states that obesity and overweight in adults are responsible for the 8% of the healthcare costs in the European Region; indirect costs, related to human lives, productivity and gaining lost, are more than double of direct costs for hospitalization and care.

Scientific literature shows that bariatric surgery may be a useful instrument to contrast severe obesity, by limiting its clinical and economic consequences.

However, surgery is not always sufficient to resolve obesity: in order to maximize its outcomes is necessary to encourage in patients the adoption of correct dietary habits, by reducing fat and sugar intake and increasing fruit and vegetables assumption, and the increase of physical activity levels, to control weight. These strategies may integrate and remodulate the therapeutic path of obese patient, in order to improve his quality of life.

In the last decades, structured educational interventions aimed at improving unhealthy habits in bariatric patients have been implemented in the USA and in several European countries.

These interventions have evidenced the importance of adapted physical activity together with counseling after surgery in supporting patients to adopt healthy choices regarding food intake and physical activity.

Employing a multidisciplinary intervention as an instrument to promote health may be considered a real health technology and be evaluated. In this case the intervention can be assessed by an

economic perspective in order to evaluate its sustainability and the achievable savings through a health technology assessment approach.

Subjects

Adult obese patients undergoing bariatric surgery with or without comorbidities, who do not refer psychological or physical limitations to physical activity.

Objectives

To design an integrated exercise-based motivational and educational path to promote lifestyle changes in bariatric patients.

Specific aims:

- communicate the importance of physical activity in the therapeutic path;
- provide information about nutrition in order to make patients aware of unhealthy habits;
- improve the adherence to the guidelines of mediterranean diet;
- provide useful instruments for weight control;
- improve self-esteem and self-efficacy to promote the ability of the subjects in taking the right decisions for their own health;
- address patients to exercise and motivate them to increase their compliance;
- improve health and health perception by promoting the adoption of healthy behaviors;
- evaluate economic benefits deriving by this multidisciplinary intervention.

Materials and methods

Bariatric patients will be invited no more than six months after surgery.

Those who will agreed to participate will be asked to give their informed consent (Attachment 1).

Patients will be voluntarily allocated in two groups: the intervention group, which will be addressed to the integrated path of exercise (adapted moderate-to-vigorous physical activity for 2-3 60 min. sessions per week) and educational/motivational support, and the control group, which will undergo Treatment As Usual (TAU). Patients will be recruited progressively. The minimum sample size is 6 (intervention/control ratio 1:2) to reach a power of 90% with a 95%IC.

Participants to the intervention will be asked to provide a medical certification for physical conditions.

The exercise program will consist of 60-minute training sessions carried out two times per week and supervised by exercise specialists with expertise in adapted physical activity. Training protocols will be tailored to participants' conditions; the intensity of exercise will be periodically increased

according to the advances of the subjects. Each session will be composed by five phases: warm-up (10 min) including continuous walking or marching and exercises for joint activation; aerobic training (25 min) consisting of moderate and high-intensity brisk walking targeted at the level 4 of the Borg's scale; exercises (15 min) to enhance strength of lower and upper limbs at 70-85% of repetition maximum (2 exercises, 3 sets of 12 repetitions); cool-down phase with agility/balance exercises (5 min) and flexibility static and dynamic exercises (5 min).

As for the motivational support, it will be performed through periodical series of bi-weekly group meetings lasting 90 minutes and guided by a psychologist with expertise on motivational interviewing for behavior change. The first sessions will be focused on the reciprocal introduction of participants and on their readiness for change; subsequently their knowledge, attitudes and beliefs regarding PA and diet will be explored.

The nutritional program will be conducted through monthly group meetings lasting 90 minutes with a trained nutritionist. It will be structured in three moments:

- the assessment of the nutritional habits of participants
- the discussion of the effects of diet on weight management
- the proposal of healthy food choices and solutions to manage nutrition through an adequate daily distribution of meals and nutrients.

Before to starting the activities in participants will be evaluated:

- physical conditions, through the assessment of blood pressure, anthropometric indexes, drug use and blood levels of glucose, glycated haemoglobin, triglycerides, cholesterol;
- psychological conditions, through the use of the Binge Eating Scale (BES) to assess the presence of binge eating disorder, of the Obesity Related Well-Being (ORWELL 97) questionnaire to evaluate quality of life, and of the Dropout Risk Profile (DRP) questionnaire to explore the motivation for behavioral change;
- nutrition, through the calculation of individual energy balance by analyzing food intake and body composition, together with daily energy requirements;
- physical activity levels, through the International Physical Activity Questionnaire;
- aerobic capacity (Test di Rockport), perceived exertion (Borg's scale), upper (dynamometer) and lower (Squat Test) limbs strength, flexibility of shoulder, elbow, ankle and knee (Range of Motion through a goniometer);
- disability deriving from back pain, through the Oswestry Low Back Pain Disability Questionnaire (OLBPDQ);
- foot posture, through the use of carbon paper.

These measurements will be repeated after 12 months since the start of the intervention.

The physical and psychological outcomes obtained from the two groups will be compared to evaluate the effectiveness of the multidisciplinary intervention. A health technology assessment will be carried out to evaluate its economic benefits.

Statistical analyses

For each group, mean values ± SD of continuous variables at the start and at the end of the intervention will be calculated. These values will be compared through the Student's *t* test for repeated measures, while the comparison between the two groups will be made through ANCOVA. Categorical variables will be analyzed through the chi squared test.

The significance level assumed will be p=0.05.

Timing

The intervention will last 12 months. At least 2 months before and 2 months after will be required for patients' recruitment and data collection and analysis.

Required resources

At least a psychologist, a nutritionist and two movement science experts will be needed, on the basis of participants' number.

A facility with a room for group meetings and a room equipped for physical activity is also required.

Expected results

We expected significant differences in surgery outcomes between intervention and TAU groups.

The considered outcomes will be:

- anthropometric measures
- quality of life and binge eating disorder
- dietary habits
- physical activity levels
- aerobic capacity, limbs strength, flexibility, disability and feet posture
- achievable savings.

Expected issues

Low compliance of participants to the intervention and drop-out at follow-up.

References

1. World Health Organization. Global status report on noncommunicable diseases 2014. WHO press, Geneva 2014.
2. Conferenza Ministeriale della Regione Europea dell'OMS, Istanbul, Novembre 2006.
3. Società Italiana di Chirurgia dell'Obesità e delle malattie metaboliche (SICOB). *Linee guida di chirurgia dell'obesità.* SICOB 2008.
4. Clinical Evidence, edizione italiana n.1, 2001: Prevenzione primaria, pag 48-74.
5. Jacobi D, Ciangur C, Couet C, Oppert JM. Physical activity and weight loss following bariatric surgery, 2010, Obesity reviews, International Association for the Study of Obesity, 12,366-377.
6. McInnis KJ, Franklin BA, Rippe JM. Counseling for Physical Activity in overweight and obese patients. Am Fam Phys 2003;67:1249-56**.**
7. King WC, Bond DS. The Importance of Pre and Postoperative Physical Activity Counseling in Bariatric Surgery, 2012 Jun 15.
8. Livhits M, Mercado C, Yermilov I, et al, Exercise Following Bariatric Surgery: Systematic Review, 2010, Obesity Surgery, 20, 657-665.
9. Delgado Floody P, Navarrete F.C, Mayorga D.J, Jara C.C, Campillo6 R.R, Poblete A.O. Hormazábal M.A Lepeley N.T, Mansilla C.S, Efectos de un programa de tratamiento multidisciplinar en obesos mórbidos y obesos con comorbilidades candidatos a cirugía bariátrica, Nutr Hosp. 2015; 31 (5): 2011-2016.
10. American College of Sports Medicine. *ACSM's Guidelines for Exercise Testing and Prescription 10th ed.* Philadelphia, PA: Wolters Kluwer; 2017.
11. Borg GA. Psychophysical bases of perceived exertion. Med Sci Sports Exerc. 1982; 14: 377–81.
12. Grupski AE, Hood MM, Hall BJ, Azarbad L, Fitzpatrick SL, Corsica JA. Examining the Binge Eating Scale in screening for binge eating disorder in bariatric surgery candidates. Obes Surg 2013;23:1-6.
13. Craig, C.L.; Marshall, A.L.; Sjöström, M.; Bauman, A.E.; Booth, M.L.; Ainsworth, B.E.; Pratt, M.; Ekelund, U.; Yngve, A.; Sallis, J.F.; et al. International physical activity questionnaire: 12-country reliability and validity. Med. Sci. Sports Exerc. 2003;35:1381–1395.
14. Mannucci E, Ricca V, Barciulli E, Di Bernardo M, Travaglini R, Cabras PL, Rotella CM. Quality of life and overweight: the obesity related well-being (Orwell 97) questionnaire. Addict Behav 1999;24:345-57.

UNIVERSITÀ DEGLI STUDI DI NAPOLI PARTHENOPE
DIPARTIMENTO DI SCIENZE MOTORIE E DEL BENESSERE

PROGETTO: PERCORSO INTEGRATO DI SUPPORTO MOTORIO-NUTRIZIONALE E ATTIVITÀ FISICA ADATTATA COME STRUMENTO TERAPEUTICO IN SOGGETTI OBESI SOTTOPOSTI AD INTERVENTI DI CHIRURGIA BARIATRICA

CONSENSO INFORMATO

Gentile Signora/e,
in questa Struttura è in programma un Progetto che si propone di far svolgere attività fisica adattata a soggetti come Lei, ovvero persone obese sottoposte ad interventi di chirurgia bariatrica.
Per svolgere tale Progetto abbiamo bisogno della Sua collaborazione e della Sua disponibilità.
L'attività fisica è il più importante fattore predittivo di mantenimento del calo ponderale determinato dall'intervento di chirurgia bariatrica. Inoltre, numerosi studi ribadiscono i benefici di "una vita in movimento" nella prevenzione primaria e secondaria di numerose malattie croniche (cardiocircolatorie, metaboliche, respiratorie, ecc), inclusa l'obesità.
Lo scopo di questo studio è divulgare la cultura della prevenzione e della promozione della salute attraverso uno stile di vita attivo, appropriato all'età e alle condizioni psicofisiche di ciascun individuo.
Il Progetto, che avrà la durata di 12 mesi prevede:

- Somministrazione di questionari:

- all'inizio del programma motorio (T_0),
- a cadenza periodica (3 mesi);
- alla fine dell'intervento motorio (T_1).

- Rilevazione del peso e delle circonferenze corporee e valutazione delle capacità fisiche:

- a tempo zero (T_0);
- alla fine dell'intervento motorio (T_1).

- Incontri di Counseling Psicologico: a cadenza quindicinale della durata di 90'.
- Incontri di Counseling Nutrizionale: a cadenza mensile della durata di 90'.
- Programma di Attività Fisica Adattata (AFA): 2 sedute settimanali della durata di 60'.

La partecipazione a questo Progetto NON comporterà alcun rischio per la sua salute, né alcun aggravio di spese a suo carico.
La Sua adesione è completamente volontaria e potrà ritirare il Consenso alla partecipazione in qualsiasi momento.
Per coloro che non prenderanno parte al progetto in maniera diretta, potrà essere richiesta la disponibilità ad una intervista telefonica, nel corso della quale verranno richieste informazioni sullo stato di salute percepito.
Ai sensi del Decreto Legge N°196/03 (Art.7 e 13) relativo alla tutela della persona per il trattamento dei dati personali, La informiamo che i Suoi dati personali verranno raccolti ed archiviati in modo adeguato e saranno utilizzati esclusivamente in modo aggregato ed anonimo per scopi di ricerca scientifica.

DICHIARAZIONE di CONSENSO

Io sottoscritto ____ residente a ____________________________

in Via ___________________________________ tel. ________________________

- dichiaro di avere ricevuto esaurienti spiegazioni in merito alla richiesta della Mia partecipazione al Progetto.
- dichiaro di aver potuto discutere tali spiegazioni, di aver potuto porre domande e di avere ricevuto risposte in merito soddisfacenti.

- accetto quindi liberamente di partecipare al Progetto, avendo perfettamente compreso tutte le informazioni sopra riportate.
- sono inoltre consapevole che secondo il rispetto della normativa vigente i Miei dati personali saranno utilizzati esclusivamente per scopi di ricerca scientifica.

Data ________________________ Firma _______________________________
